# A new species of spider of the genus *Sadala* Simon, 1880 (Araneae, Sparassidae) from the Yasuni Biosphere Reserve, Amazonian lowlands of Ecuador

**DOI:** 10.1002/ece3.10242

**Published:** 2023-07-06

**Authors:** Pedro Peñaherrera‐R., Diego F. Cisneros‐Heredia

**Affiliations:** ^1^ Colegio de Ciencias Biológicas y Ambientales COCIBA, Instituto de Biodiversidad Tropical IBIOTROP, Laboratorio de Zoología Terrestre, Museo de Zoología Universidad San Francisco de Quito USFQ Quito Ecuador; ^2^ Tiputini Biodiversity Station Universidad San Francisco de Quito USFQ Quito Ecuador; ^3^ Instituto Nacional de Biodiversidad INABIO Quito Ecuador

**Keywords:** giant crab spider, huntsman spider, new species, northern South America, taxonomy, Tiputini

## Abstract

We describe a new species of giant crab spider of the genus *Sadala* Simon, 1880 collected in Lowland Evergreen rainforests at the Tiputini Biodiversity Station, Yasuni Biosphere Reserve, Amazonian Ecuador. This new species corresponds to the first record of the genus from Ecuador. Females of the new species of *Sadala* are similar to *S. punicea* and *S*. *nanay*, by having the epigyne with a median septum diamond‐shaped posteriorly. The new species is easily distinguished from *S. punicea* and *S*. *nanay* by having relatively straight anterior lateral margins of the median septum. This study increases to 10 the number of described species of *Sadala*.

## Graphical abstract

The first species of spider of the genus *Sadala* is described from Ecuador. The new species was discovered at the Tiputini Biodiversity Station, a research station in the Yasuni Biosphere Reserve, Amazonian lowlands of Ecuador.

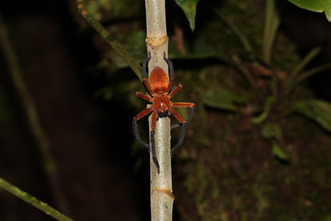

## Resumen

Describimos una nueva especie de araña cangrejo gigante del género *Sadala* Simon, 1880 colectada en Bosque Lluvioso Siempreverde de Tierras Bajas en la Estación de Biodiversidad Tiputini, Reserva de la Biósfera Yasuní, Amazonía del Ecuador. Esta nueva especie corresponde al primer registro del género en Ecuador. Las hembras de la nueva especie de *Sadala* se diferencian de todos sus congéneres, excepto *S. punicea* y *S*. *nanay*, por un septo medio del epiginio con forma de diamante posteriormente. La nueva especie se diferencia fácilmente de *S. punicea* y *S*. *nanay* por tener los bordes anterolaterales del septo medio relativamente más rectos. Este estudio incrementa a diez el número de especies descritas de *Sadala*.

## 1 INTRODUCTION

The genus *Sadala* Simon, 1880 (family Sparassidae) was first proposed to include 12 species (including some originally placed in *Sparassus* Walckenaer, 1805 and *Olios* Walckenaer, 1837) (Rheims & Jäger, 2022; Simon, 1880). However, *Sadala* was later synonymised with *Sparassus* by Simon (1897) and then included in *Olios* by Simon (1903). Rheims and Jäger (2022) revalidated the genus *Sadala* to include nine species: *Sadala keyserlingi* Simon, 1880 (type species); *S. kaiabi* Rheims & Jäger, 2022; *S. nanay* Rheims & Jäger, 2022; *S. nigristernis* Simon, 1880; *S. punicea* Simon, 1880; *S. rufa* (Keyserling, 1880), *S. tabatinga* Rheims & Jäger, 2022; *S. velox* Simon, 1880; and *S. yuyapichis* Rheims & Jäger, 2022.

Spiders of the genus *Sadala* are sparassids with 10.3–24.3 mm in total length (Rheims & Jäger, 2022). Species of *Sadala* are distributed in Central and South America, from Panama to central‐west Brazil (Rheims & Jäger, 2022). Although there are no species of *Sadala* described from Ecuador, *S. rufa* may be present in the country since the type specimens of one of its synonyms were allegedly obtained in Ecuador (*Olios corallinus* Schmidt, 1971). Also, Rheims & Jäger (2022, figures 1 and 3) presented photographs of unidentified *Sadala* from the Amazonian lowlands of Ecuador.

Recent expeditions to the Tiputini Biodiversity Station, a research station in the Yasuni Biosphere Reserve, Amazonian lowlands of Ecuador, resulted in the collection of an undescribed species of *Sadala*, which we described herein.

## 2 MATERIALS AND METHODS

We carried out fieldwork at the Tiputini Biodiversity Station (TBS, 0°38′13″ S, 76°08′59″ W, 230 m elevation), ca. 280 km ESE from Quito, province of Orellana, Republic of Ecuador. TBS is a research station founded in 1994 by Universidad San Francisco de Quito USFQ on a 744‐ha tract of undisturbed Lowland Evergreen Forest on the northern bank of the Tiputini, within the Yasuni Biosphere Reserve, one of the most biodiverse regions in the world (Bass et al., 2010; Blake et al., 2012; Cisneros‐Heredia, 2003, 2006; Romo et al., 2017; Ryder & Sillett, 2016). The station contains a variety of habitats, including non‐flooded forests (Terra Firme), flooded forests (Várzea and small areas of Igapo and Palm swamps), and natural GAPs. Mean annual precipitation is about 2700–3100 mm, and climate is relatively aseasonal, but peak rainfall occurs from April to August, and the driest months are August and November to March (Blake et al., 2012; Cisneros‐Heredia, 2003, 2006; Romo et al., 2017; Ryder & Sillett, 2016).

Opportunistic collections were conducted at night on trails in Terra Firme and Varzea forests at TBS. Specimens were collected by hand, transported to the laboratory in plastic containers with leaf litter, photographed alive, and euthanized with direct intra‐cardiac delivery of potassium chloride KCl, following protocols described by Bennie et al. (2012). Specimens were preserved in 75% ethanol. Female genitalia were excised using a syringe tip, soft tissue was digested with a solution of 15% potassium hydroxide KOH and washed in distilled water and 75% ethanol. Epigyne and vulva are preserved in 75% ethanol in small vials stored together with each specimen. Examined specimens are deposited at the Museo de Zoología, Universidad San Francisco de Quito (ZSFQ), Quito, Ecuador. Information on species for comparative diagnoses was obtained from the recent comprehensive revision of *Sadala* by Rheims and Jäger (2022).

Specimens were examined and measured under an Olympus SZX16 stereomicroscope with an Olympus DP73 digital camera. Measurements were recorded with Micro Imaging Software CellSens for Olympus. All measurements are presented in millimeters. Chelicerae length is considered in total length. Description, definitions, terminology, and measurements follow the standards and format proposed by Rheims and Jäger (2022). Leg spinnation description follows definitions and terminology proposed by Petrunkevitch (1925). Abbreviations are as follows for legs spinnation: d, dorsal; p, prolateral; r, retrolateral; v, ventral; and female genitalia: CO, copulatory opening; FD, fertilization duct; ft, first turn of duct system; GP, glandular projection; LL, lateral lobe; MS, median septum; TP, triangular projection; SP, spermathecae. Life colors are described based on photographs of live spiders taken in the field. The adjective “pectinated” describes a coloration pattern having marks shaped like branches of a comb (Maggenti et al., 2017).

We obtained occurrence data for individuals of *Sadala* from mainland Ecuador updated to iNaturalist (https://www.inaturalist.org), a citizen science platform by the California Academy of Science and National Geographic. Data search and extraction were conducted in July 2022. For each occurrence point, we compiled geographic data and all other associated information, and localities were reviewed and validated individually, following protocols described by Cisneros‐Heredia and Peñaherrera‐Romero (2020) and Cisneros‐Heredia et al. (2022). Geographic records of *Sadala* from mainland Ecuador used for this paper are available in Figshare: https://doi.org/10.6084/m9.figshare.22337647.

## 3 RESULTS

### 3.1 *Sadala rauli* Peñaherrera‐R. & Cisneros‐Heredia, new species (Figures 1, 2, 3, 4).

**FIGURE 1:**
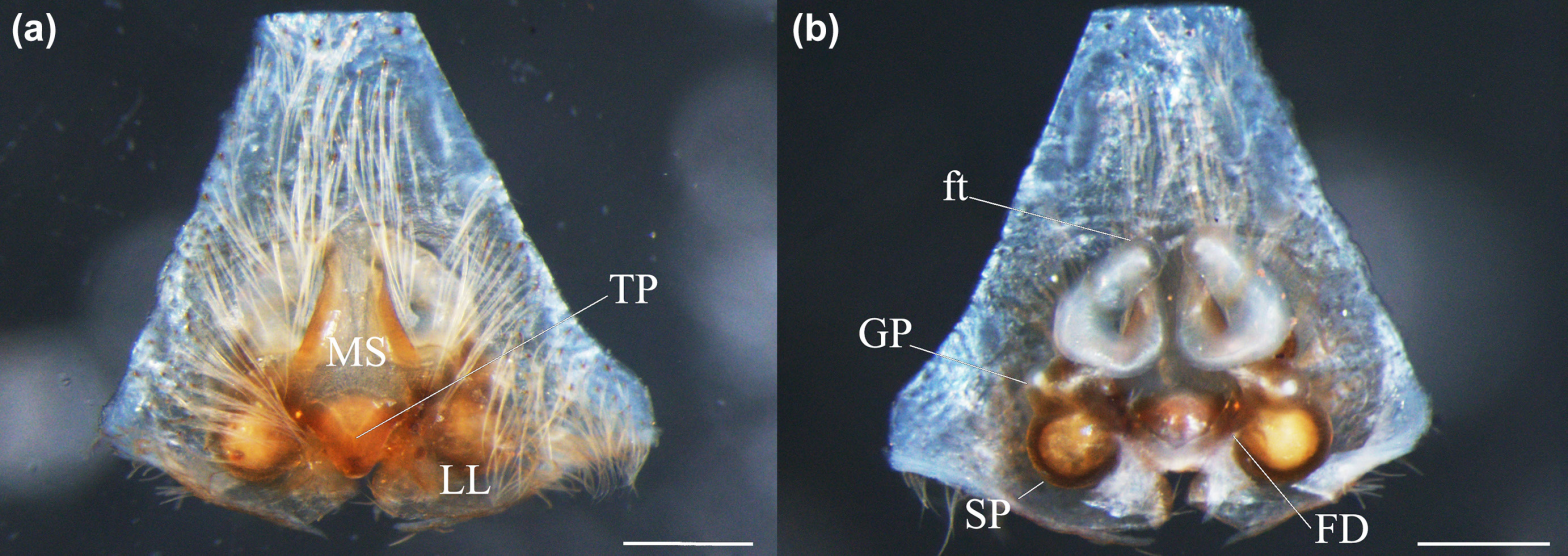
Holotype of *Sadala rauli* sp. nov., female (ZSFQ‐i8258). (a) Epigyne, (b) vulva. Scale lines: 0.4 mm.

**FIGURE 2:**
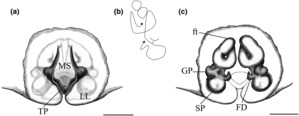
*Sadala rauli* sp. nov., female (ZSFQ‐i8258). Illustrations of the (a) epigyne, (b) schematic course of the internal duct system, (c) vulva.

**FIGURE 3:**
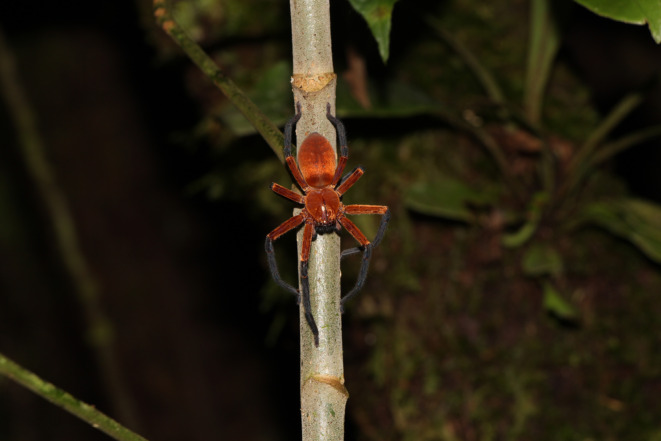
Habitus of *Sadala rauli* sp. nov., female paratype, at the Tiputini Biodiversity Station, province of Orellana, Ecuador.

**FIGURE 4:**
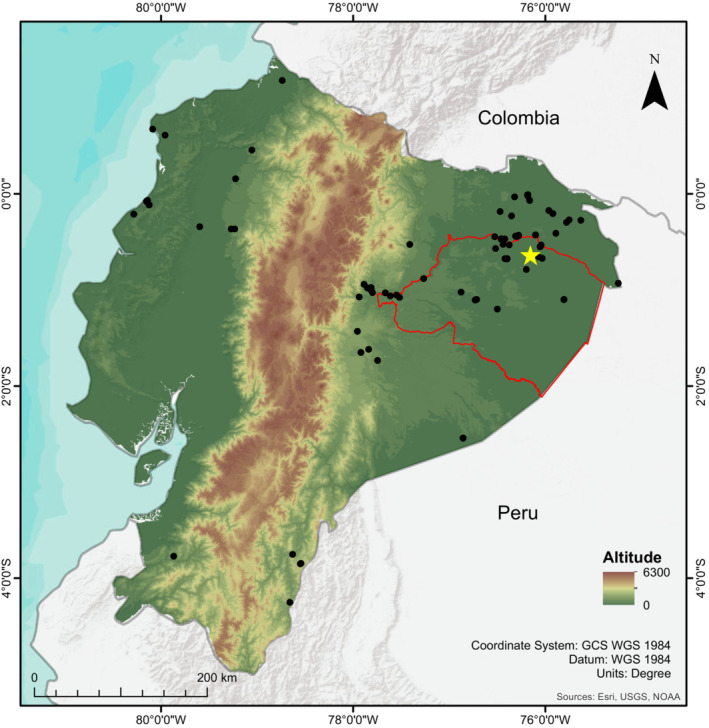
Records of *Sadala* in Ecuador. Yellow star = type locality of *Sadala rauli* sp. nov., Tiputini Biodiversity Station, province of Orellana. Black dots = Citizen science records of *Sadala* obtained from iNaturalist. Red perimeter = Yasuni Biosphere Reserve.

**Holotype.** ZSFQ‐i8258, adult female collected at the Tiputini Biodiversity Station, Guacamayo trail (0°63′78″ S, 76°15′50″ W, 222 m elevation), provincia de Orellana, República del Ecuador, 30 May 2022, P. Peñaherrera‐R., R. J. León‐E., A. Guerrero‐Campoverde, D. F. Cisneros‐Heredia leg.

**Paratypes.** ZSFQ‐i8259, same data as holotype but collected at Matapalo trail on 29 May 2022; ZSFQ‐i8260 at Guacamayo trail on 02 July 2022.

**Etymology.** The specific name is a patronym for Victor Raul Peñaherrera de la Cadena, grandfather of Pedro Peñaherrera‐R., who in life had the project of narrating the history of Ecuador in a book that could not be completed and now will form part of the natural history of Ecuador immortalized in this species.

**Generic placement.** The new species is assigned to the genus *Sadala* by having chelicerae with three promarginal teeth and intermarginal denticles, 5–10 escort setae at the base of fang, short‐toothed female palpal claw, three pairs of spines on ventral tibiae I–II, and median septum of epigyne with triangular scape‐like projection (Rheims & Jäger, 2022).

**Diagnosis.** Females of *Sadala rauli* sp. nov. resembles *S. punicea* and *S*. *nanay* by having the epigyne with a long TP, wider than long, and MS diamond‐shaped posteriorly (Figures 1 and 2). The new species is easily differentiated from *S. punicea* and *S*. *nanay* by having relatively straight anterior lateral margins of the MS (more curved in *S. punicea* and *S. nanay*). Also, *S. rauli* differs from *S. punicea* by having a MS more than 1.5 times longer than wide with elliptical CO.

**Description of holotype.** Female holotype: Total length 21.62. Cephalothorax: 7.44 long, 7.36 wide. Abdomen: 11.75 long, 7.94 wide. Eyes diameters: 0.47, 0.37, 0.31, 0.35; interdistance: 0.43, 0.45, 0.80, 0.86, 0.47, 0.56. Legs: I (8.70/3.44/7.51/8.33/2.17); II (9.49/3.35/8.87/8.70/2.13); III (7.24/3.08/5.82/5.39/1.83); IV (8.24/2.90/6.39/6.96/2.14). Spination follows the generic pattern except for patellae I–II: p0‐1‐0, v0, d0, r0‐1‐0; III: p0‐2‐0, v0, d0, r0. Epigyne: EF as long as wide; MAB embedded (cleared during soft tissue digestion); TP wider than long; LL closer to each other posteriorly (Figures 1 and 2). Vulva: FW slender; GP globose, wider than longer, arising from duct close to SP; rounded; FD anteriad (Figures 1 and 2).

**Coloration.** Cephalothorax and abdomen orange‐brown, dark brown stripe extending posteriorly from between median eyes abdomen; with a darker pectinated pattern on abdomen; coxae, trochanters and femurs orange‐brown; patellae, tibiae, tarsi, and metatarsi black; clypeus and chelicerae black; sternum orange‐brown (Figure 3).

**Male.** Unknown.

**Variation.** Paratype females, total length: 18.94–19.46; cephalothorax length: 7.06–7.57; femur I length: 8.08–8.36. Lighter pectinated pattern on the abdomen and lighter heart mark than the holotype.

**Distribution and natural history.** Known currently only from the Tiputini Biodiversity Station in the Amazonian lowlands of Ecuador, at 2–3 m (Figure 4). Specimens were found active at night perched on vegetation 1–2 m above the floor in old‐growth Lowland Evergreen Non‐Flooded (Terra Firme) Forest (Figure 3).

**Remarks.** There are 138 observations of individuals of *Sadala* in iNaturalist (Figure 4), with 17 of these records coming from the Tiputini Biodiversity Station. The observations from Tiputini could correspond to *S. rauli* sp. nov. or other species, but it is impossible to confirm any identification without examining the genitalia. In addition, 118 observations come from different localities in the Amazonian lowlands and eastern Andean slopes of Ecuador. Interestingly there are also 16 observations of *Sadala* from the Pacific lowlands of Ecuador that could correspond to undescribed species or even *S. rufa*.

## 4 DISCUSSION

*Sadala rauli* sp. n. is the first species of the genus from Ecuador, increasing to 10 the number of described species of *Sadala*. Six species of *Sadala* have been reported from western Amazonia: *S. nanay*, *S. nigristernis*, *S. punicea*, *S. rauli* sp. n., *S. tabatinga*, *S. velox*, and *S. yuyapichis* (Rheims & Jäger, 2022). Two of them have been found in sympatry, at the Yuyapichis River, department of Huanuco, Peru (*S. velox* and *S. yuyapichis*), while five others are known from nearby localities in the department of Iquitos, Peru, and the state of Amazonas, Brazil (*S. nigristernis*, *S. nanay*, *S. punicea*, *S. velox*, *S. tabatinga*) (Rheims & Jäger, 2022). The similarities between *S. rauli*, *S. punicea* and *S. nanay* could point to a close evolutionary relationship between them, and phylogenetic studies are required to confirm this hypothesis. The number of observations available in iNaturalist suggests that there could be additional localities of *S. rauli* or other, possibly undescribed, species of *Sadala* in Ecuador, probably with some localities holding more than one species. Additional collections and studies are required to determine the species richness and distribution of *Sadala* across Ecuador, which seems to be underestimated.

## AUTHOR CONTRIBUTIONS

**Pedro Peñaherrera‐R.:** Conceptualization (equal); data curation (equal); formal analysis (equal); investigation (equal); methodology (lead); project administration (supporting); software (equal); validation (equal); visualization (equal); writing – original draft (equal); writing – review and editing (equal). **Diego F. Cisneros‐Heredia:** Conceptualization (equal); data curation (equal); formal analysis (equal); funding acquisition (lead); investigation (equal); methodology (supporting); project administration (lead); resources (lead); software (equal); supervision (lead); validation (equal); visualization (equal); writing – original draft (equal); writing – review and editing (equal).

## FUNDING INFORMATION

Universidad San Francisco de Quito USFQ and Tiputini Biodiversity Station supported this work through operative funds assigned to the Institute of Tropical Biodiversity IBIOTROP and research and outreach funds assigned to Diego F. Cisneros‐Heredia.

## CONFLICT OF INTEREST STATEMENT

None.

## Data Availability

The data that support the findings of this study are openly available in figshare: 10.6084/m9.figshare.22337647.
